# A Multiview Model for Detecting the Inappropriate Use of Prescription Medication: Machine Learning Approach

**DOI:** 10.2196/16312

**Published:** 2020-07-06

**Authors:** Lin Zhuo, Yinchu Cheng, Shaoqin Liu, Yu Yang, Shuang Tang, Jiancun Zhen, Junfeng Zhao, Siyan Zhan

**Affiliations:** 1 Research Center of Clinical Epidemiology Peking University Third Hospital Beijing China; 2 Department of Epidemiology and Biostatistics School of Public Health Peking University Beijing China; 3 Department of Pharmacy Peking University Third Hospital Beijing China; 4 School of Electronics Engineering and Computer Science Peking University Beijing China; 5 Center for Data Science in Medicine and Health Peking University Beijing China; 6 Department of Pharmacy Ji Shui Tan Hospital and Fourth Medical College of Peking University Beijing China

**Keywords:** inappropriate use of prescription medication, topic model, latent Dirichlet allocation, multiview learning, prescription review

## Abstract

**Background:**

The inappropriate use of prescription medication has recently garnered worldwide attention, but most national policies do not effectively provide for early detection or timely intervention.

**Objective:**

This study aimed to develop and assess the validity of a model that can detect the inappropriate use of prescription medication. This effort combines a multiview and topic matching method. The study also assessed the validity of this approach.

**Methods:**

A multiview extension of the latent Dirichlet allocation algorithm for topic modeling was chosen to generate diagnosis-medication topics, with data obtained from the Chinese Monitoring Network for Rational Use of Drugs (CMNRUD) database. Topic mapping allowed for calculating the degree to which diagnoses and medications were similarly distributed and, by setting a threshold, for identifying prescription misuse. The Beijing Regional Prescription Review Database (BRPRD) database was used as the gold standard to assess the model’s validity. We also conducted a sensitivity analysis using random samples of validated prescriptions and evaluated the model’s performance.

**Results:**

A total of 44 million prescriptions were used to generate topics using the diagnoses and medications from the CMNRUD database. A random sample (15,000 prescriptions) from the BRPRD was used for validation, and it was found that the model had a sensitivity of 81.8%, specificity of 47.4%, positive-predictive value of 14.5%, and negative-predictive value of 96.0%. The model showed superior stability under different sampling proportions.

**Conclusions:**

A method that combines multiview topic modeling and topic matching can detect the inappropriate use of prescription medication. This model, which has mediocre specificity and moderate sensitivity, can be used as a primary screening tool and will likely complement and improve the process of manually reviewing prescriptions.

## Introduction

It is estimated that more than 50% of medicines are inappropriately prescribed, dispensed, or sold, which represents a universal challenge for medical practice [1]. Furthermore, in developing countries, the treatment of about 60% to 70% of patients in primary care does not meet standard treatment guidelines [2]. This inappropriate use of prescription is wasteful and costly, and can increase the risk of adverse drug reactions [3]. Finally, the overuse of antimicrobial and antibiotic injections may result in certain pathogens developing antibiotic resistance [4].

The excessive use of antibiotics is common in China, as is the injection of traditional Chinese medicines [5-7]. Antibiotics are present in 50% of prescriptions and injectable medicines in 30%, exceeding the World Health Organization’s standard treatment guidelines [7]. The Chinese government released the *Management Practices of Hospital Prescription Comment (Trial)* in 2010 to assess compliance with rational criteria for using prescription drugs [8]. This document requires that each hospital assign trained pharmacists monthly to review a minimum of 100 randomly sampled prescriptions. However, these reviews are currently associated with limited coverage, high omission rates, a lack of representativeness, and supervisory lag. All of this points to the urgency of improving the review process, especially when Chinese hospitals are witnessing a continuous daily increase in outpatient prescriptions [9,10].

A few knowledge-based approaches have been implemented in the health care information systems (HISs) of Chinese hospitals to screen the appropriateness of prescriptions [11,12]. Prior knowledge, including treatment guidelines, formularies, package inserts, expert knowledge, and published literature, indicates that these systems are generally working well. However, they are time-consuming and costly to establish and maintain [13]. Furthermore, timely updates to these systems are challenging because of the continuous availability of both new drugs and new research.

Currently, both supervised and unsupervised data-driven methodologies, whether as alternatives or supplements to the systems listed above, are being used to identify outliers and detect inappropriate prescriptions. Such approaches remain constrained, however, by the difficulty associated with using supervised methods to obtain high-quality labeled sample data [14,15]. Other limiting factors include defining outliers and considering their association rules, which effectively account for the relationships between features [16]. Usually, diagnosis and medication are closely and consistently related to the clinical condition of the patient. In the absence of this consistency, prescriptions are more likely to be inappropriate (or anomalous). Contextual anomaly detection is one approach to capture the relationship between features (eg, between medication and diagnosis) and to detect exceptions caused by feature mismatch [17,18]. Nonetheless, this does not work well with prescription data or similar information in a high-dimensional sparse space [19].

By contrast, a topic modeling method, the latent Dirichlet allocation (LDA) method [20], has been proven to be useful in dimensional reduction when mining patient records [21]. Here, a “topic” is defined as a collection of semantically related terms that appear frequently and relate to a common subject [22]. LDA, a probabilistic statistical model with the assumption that topic distributions are drawn from their prior distributions, can be used to describe the composition of high-dimensional unstructured text and to capture clusters of words that reveal critical concepts [21,23]. Beginning with its appearance in the biomedical domain, LDA has been used in mining clinical pathway patterns [24-26], image processing [27-29], risk stratification [30], and bioinformatics [31-33]. One drawback of LDA is that it cannot simultaneously consider both diagnosis and medication. We therefore adopted a multiview [23,34-36] concept that enhances the topic modeling capacity of LDA and coordinated it with anomaly detection techniques to build a multiview LDA model (MV-LDA). This model, which was tested in our previous simulation study, had a greater area under the precision-recall curve [37] than the two traditional methods (point anomaly detection and contextual anomaly detection) and had better suitability for high-dimensional sparse data.

## Methods

### Data Sources

One subsystem of the Chinese Monitoring Network for the Rational Use of Drugs (CMNRUD) was the data source for model development. The CMNRUD was launched by the Chinese Ministry of Health in 2010, and it covers over 86% (30/35) of the provinces in China [38], including 60% of the nation’s tertiary hospitals (955 hospitals) and 6% of its secondary hospitals (375 hospitals) [39]. Each monitoring hospital must upload encrypted data every month. The system organizes the prescriptions in a stipulated uniform structure, and thereafter, some of the cleaned data are checked by data management professionals. Since 2013, the system has been performing automatic uploading, preliminary cleaning, recoding, and verification.

The CMNRUD consists of the following four monitoring subsystems: outpatient prescriptions, clinical drug use, medical damage, and critical disease. Anonymized data from outpatient prescriptions (from October to December 2016) were used to build the model for detecting prescription misuse. These data include demographic, diagnostic, and drug-related information. Diagnostic information includes the patient ID, diagnosis date, diagnosis description, and diagnostic code (10th revision of the International Classification of Diseases, ICD-10), which are directly related to the purpose of the patient’s visit or their condition. It sometimes may not include other complications that do not require further treatment. A higher diagnosis ranking was associated with more visit relevance. Available drug-related information (no more than five medications per prescription) includes information such as the prescription date, generic and brand names, corresponding Anatomical Therapeutic Chemical code, dosage, and administration route. The medications were listed randomly, preventing them from being mapped to the corresponding diagnoses on a one-to-one basis. The variables taken from the CMNRUD are presented in Table 1.

The data for model validation were randomly selected from the Beijing Regional Prescription Review Database (BRPRD) [40], which was created by the Beijing Municipal Administration of Hospitals in 2010. The BRPRD extracts 1 week of prescriptions every quarter from the HISs of 17 tertiary hospitals and five secondary hospitals in Beijing. A total of 19 hospitals from the BRPRD were included among the 65 CMNRUD monitoring hospitals from Beijing and accounted for 5.4% (19/349) of the hospitals in the entire CMNRUD database. The prescription variables include treatment type, prescription number, prescription date, age, sex, diagnosis, and medication. As part of the standard procedure, a prescription review board of trained clinicians and clinical pharmacists regularly examines the prescriptions individually based on a standardized guideline and then captures inappropriate data in the BRPRD database [8,41].

**Table 1:** Main variables in the Chinese Monitoring Network for the Rational Use of Drugs outpatient prescription monitoring subsystem.

Information	Variables^a^
Basic information (patients)	Patient ID, treatment card number, sex, age, and age range
Basic information (hospital, department, and doctor)	Hospital name, hospital grade, hospital type, region, department, and doctor ID
Diagnosis	Diagnosis name, and ICD-10^b^ by class, suborder, and type
Medication^c^	Prescription ID, prescription type, prescription date, ATC^d^ code, drug trade name, drug generic name, specification, quantity, unit, dosage, usage, price, pharmaceutical company, and individual hospital information

^a^The variables indicate the features of the multiview latent Dirichlet allocation model.

^b^ICD-10: 10th revision of the International Classification of Diseases.

^c^Since a generic medicine works the same as its branded version and owing to the limitation of the Anatomical Therapeutic Chemical’s lack of codes for traditional Chinese medicine, we used generic names, which are well recorded in the database, to build the topic model.

^d^ATC: Anatomical Therapeutic Chemical.

### Study Approval

The Institutional Review Board of Peking University reviewed and approved the study protocol before the study commenced, and it determined that informed consent was not required (reference number: IRB00001052-17003-Exempt).

### Study Design

We developed and evaluated an MV-LDA model for detecting the inappropriate use of prescription medications in the following four steps: (1) data preparation, gathering and cleaning data from the CMNRUD and BRPRD database; (2) topic generation, using MV-LDA topic-modeling methods and CMNRUD data to extract associations between diagnoses and medications; (3) inferring and anomaly scoring, using BRPRD data and the topics extracted in step 2 to infer the distribution of each prescription and measuring the degree to which diagnoses and medications show similar distributions (less similarity is associated with more likelihood that the item represents the inappropriate use of prescription medication); (4) model evaluation and sensitivity analysis, evaluating the model for detecting prescription misuse with the results of the BRPRD review.

#### Step 1: Data Preparation

Prescriptions between October 2016 and December 2016 from CMNRUD were used, and those with missing prescription identifiers or with medication withdrawal were excluded. The patient ID, treatment card number, prescription date, diagnosis name, and generic drug name were chosen to build the topics.

For model evaluation, considering the sensitivity and specificity (71.5% and 68.8%, respectively) of the Apriori algorithm in previous work [42,43], we set both the expected sensitivity and specificity at 80%. By setting a significance level of .05 and an allowable error of 0.05, we needed at least 14,471 prescriptions given 1.7% prevalence [44] of prescription misuse according to equation 1.


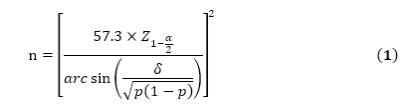


Finally, we randomly selected a total of 15,000 prescriptions from 2016 BRPRD data that had already been manually reviewed by experienced pharmacists, with the prescriptions that included the following three variables: prescription ID, diagnosis, and medication.

#### Step 2: Topic Generation

The study is based on the assumption that the prescription database is mostly composed of regular instances (ie, rational appropriate prescriptions), and a probabilistic model is fitted to all features.

**Figure 1:**
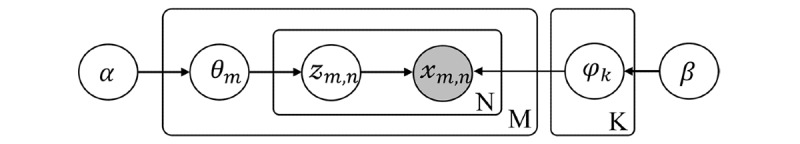
Graphical representation of the latent Dirichlet allocation model. K: number of topics; M: number of documents; N: number of words in each document; x: observed words in the document m; z: topic of nth word in a document m; θ: topic distribution for document m (document-topic distribution); φ: topic-word distribution; α: hyperparameter of θ; β: hyperparameter of φ.

LDA assumes that a set of documents or instances exhibits a specific number of latent independent topics, and then, the given topics generate the terms probabilistically. A graphical representation of the LDA model is given in Figure 1. With specific input records and setting hyperparameters *α* and *β*, LDA can detect *K* topics, formally presented as two multinomial distributions (topic-word distribution *φ* and document-topic distribution *θ*). The LDA model formula is shown in equation 2.


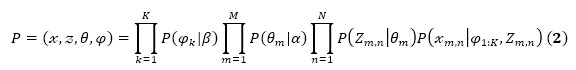


**Figure 2:**
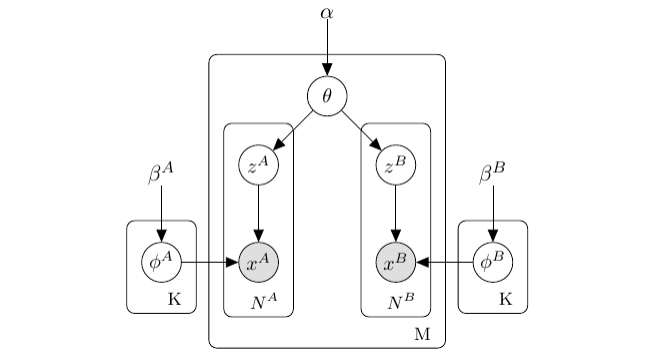
Graphical representation of the multiview latent Dirichlet allocation model. K: number of topics; M: number of prescriptions; N^A^: number of diagnoses per prescription; N^B^: number of medications per prescription; x^A^: diagnosis (type A feature); x^B^: medication (type B feature); z^A^: topic of x^A^; z^B^: topic of x^B^; φ^A^: topic-diagnosis distribution; φ^B^: topic-medication distribution; β^A^: hyperparameter of φ^A^; β^B^: hyperparameter of φ^B^; θ: prescription-topic distribution; α: hyperparameter of θ.

Like the standard LDA, MV-LDA can be represented as a probability pattern, as shown in Figure 2. In the medical domain, we can consider a clinical condition (topic) as a probability distribution over related diagnostic codes, and a patient’s diagnostic record can be regarded as a “document” composed of different clinical topics. The same applies to medication. In our study, we used CMNRUD data to generate MV-LDA topics. For prescription *m*, features A and B represent the diagnoses and medications, respectively. Both follow the same generative process mentioned above (ie, they comply with the same topic distribution *θ*), and then, *α* and *β* become the hyperparameters of prescription-topic distribution and topic-diagnosis (or topic-medication) distribution within topics. Moreover, *φ^A^* and *φ^B^* represent the topic feature distribution of A and B, respectively. In summary, the MV-LDA model is a combination of two separate LDA models (here they are called *f^A^* and *f^B^*) integrated by the common distribution *θ*. In Figure 2, *N^A^* and *N^B^* are the total numbers of diagnoses and medications with each prescription; this value can only be a discrete integer.

The MV-LDA model generates topics as follows: (1) For each topic, draw features *φ^A^*~*Dirichlet(β^A^)* and draw features *φ^B^*~*Dirichlet(β^B^)*; (2) For each prescription, draw topic proportions *θ~Dirichlet(α)*; for each feature A, draw *z_m,n_~Mult(θ_m_)* and draw *x_m,n_~Mult(φ_z_^A^)*; and for each feature B, repeat the steps for feature A.

We adopted Gibbs sampling to create the model and parameters *φ* and *θ*. Topics were first randomly assigned to all of the features. Every diagnosis (type A feature) or medication (type B feature) in each prescription that corresponds to a topic is iteratively sampled. The calculation of the conditional probability of *x^A^* is shown in equation 3, with the related notations shown in Table 2. Here, the first factor of equation 3 only accounts for the type A feature topic-diagnosis counts, whereas the second factor calculates the prescription-topic for all features. This formula also applies to type B features.

The features of both types (A and B) were iteratively sampled for each prescription until the model converged. Thereafter, we utilized the result to calculate the parameters *φ^A^* and *φ^B^*, which are used for the inferring step, and *φ^A^* can be calculated as in equation 4. We set eight topic numbers (15, 20, 25, 30, 35, 40, 45, and 50) and built the MV-LDA model according to previous research and a pilot study revealing that LDA had a moderate ability in terms of generating topics for electronic medical records with a topic number of around 30 [45].


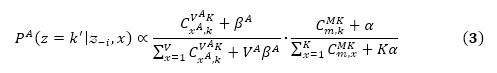



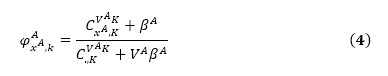


**Table 2:** Notations of the multiview latent Dirichlet allocation model in Gibbs sampling.

Variable	Description
*K*	Topic number
*V* ^A^	Number of diagnoses (type A feature)
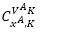	Number of times that *x*^A^ is assigned to topic *k*
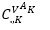	Number of times that any feature A is assigned to topic *k*
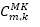	Number of all features of prescription *m* (including both A and B) assigned to topic *k*
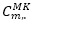	All features in prescription *m*

#### Step 3: Inferring and Anomaly Scoring

In this step, a separate dataset was used for inferring 15,000 randomly sampled prescriptions from the BRPRD (2016) that had already been manually reviewed by experienced pharmacists. Each feature in the MV-LDA model can be treated as an independent LDA model and can be inferred separately. To be specific, for the MV-LDA model obtained in the previous learning step, *φ^A^* can be used to detect the new prescriptions in question, but this only pertains to estimations of the topic distribution under feature A. The equation for this is as follows:


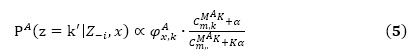


In this equation, *φ_x,k_^A^* is the value of the topic distribution under the circumstance of topic *k* and feature *x*. Finally, as in the topic generating step (step 2), we inferred the marginal *θ* based on the Gibbs sampling shown in equation 5, which indicated the proportion of feature A assigned to topic *k* for each prescription. Additionally, 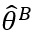 is calculated under type B features. For each test prescription, both 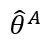 and 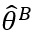 were inferred and used to calculate the anomaly score.


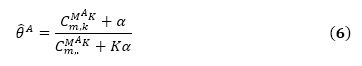


The assumption mentioned in step 2 is that the given order of diagnoses and prescribed medication should show consistency (ie, the values for 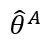 and 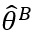 should be equal or close to each other), and if not, the prescription might be inappropriate. The similarity between 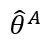 and 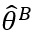 was measured using novel topic mapping (TM) methods [46]. TM was performed in the following manner: we allocated topic feature distributions from the MV-LDA model for every diagnosis or medication before matching. First, high probability topics were tagged for each diagnosis. Thereafter, we similarly identified the most probable topics for each medication and added up the total. When a topic was not tagged, it was assigned an anomaly score of 1. Finally, the anomaly scores for each prescription were summed, and different thresholds were used to filter potentially inappropriate prescriptions.

#### Step 4: Model Evaluation and Sensitivity Analysis

The same prescriptions (15,000 randomly sampled prescriptions from the BRPRD in 2016) were inferred and detected by the MV-LDA model. Multimedia Appendix 1 shows the confusion matrix of the screening test we used. The sensitivity, specificity, positive-predictive value (PPV), negative-predictive value (NPV), and Youden’s index were computed from the results to compare the assessments between the model and the experts and to identify the best performance parameter setting of TM. A sensitivity analysis was performed by randomly sampling 90%, 70%, 50%, 30%, and 10% of prescriptions from the evaluation data of the 15,000 prescriptions. The sensitivity, specificity, PPV, NPV, Youden’s index, and area under the receiver operating characteristic curve were compared. It should be noted that the overlap between training data and evaluation data was small enough to be ignored.

## Results

### Prescriptions

A total of 44,325,065 prescriptions from 22 million patients (138,535,092 records) at 349 hospitals, including 286 tertiary and 63 secondary hospitals, were used in our topic modeling process. This included 5,653 types of medications and 22,643 diseases or conditions. In the validation dataset, there were 14,166 (94.4%) outpatient prescriptions and 834 (5.6%) emergency prescriptions. Of these, 13,524 (90.2%) prescriptions satisfied the appropriate criteria (marked as “appropriate”) and 1476 (9.8%) failed (marked as “inappropriate”).

### Multiview Latent Dirichlet Allocation Topic Generation Results

By setting the topic parameters, we obtained eight topic models, all with commonly diagnosed diseases in clinical practice. For example, the model (*K*=30) included cardiovascular diseases, diabetes, chronic nephrosis, osteoporosis, and some respiratory infections. Regarding topic 27, hypertension had a 93.3% probability of appearing in this topic, and amlodipine, nifedipine, levamlodipine, and metoprolol had probabilities of 11.7%, 8.9%, 7.7%, and 6.1%, respectively. The top probability diagnoses in topic 23 were bronchitis, pneumonia, and bronchopneumonia, with the proportions of 55.6%, 21.8%, and 12.1%, respectively, whereas the corresponding medications were ambroxol (11.2%), budesonide (10.7%), azithromycin (9.9%), and terbutaline (6.2%). We also obtained topics related to gastrointestinal diseases and mental and dermal disorders. The details pertaining to the top 10 topics and their allocations are shown in Multimedia Appendix 2.

After comparing the training results of the topic models with settings at *K*=15, 20, 25, 30, 35, 40, 45, and 50 for the training results, it was found that a smaller topic number was associated with a weaker relation between the topics on one side and diagnoses and medications on the other, which were likely to appear more dispersed and had a lower probability of appearing in a topic. As the set value of the number of topics increased, the ability to summarize the disease was enhanced, that is, the subject-feature distribution of topic learning became more concentrated, the feature became more likely to appear in the topic, and the proportions of diagnosis and medication tended to be uniform.

### Multiview Latent Dirichlet Allocation Evaluation Sensitivity Analysis Results

**Figure 3:**
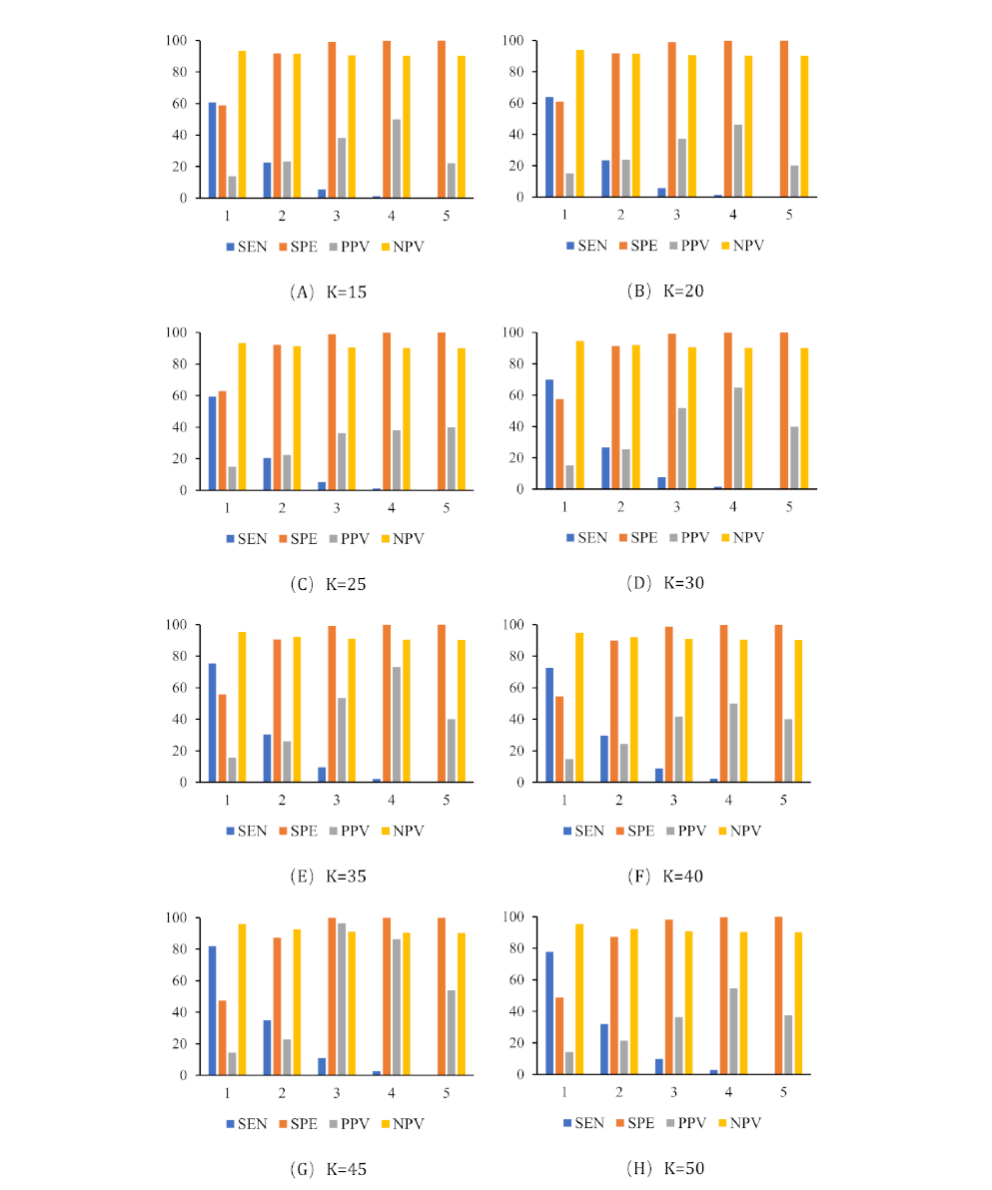
Summary of the performance of multiview latent Dirichlet allocation model with TM detection methods under different thresholds. Horizontal axis: thresholds of TM methods (from 1 to 5). Vertical axis: percentage of SEN, SPE, PPV, and NPV. K: number of topics; NPV: negative-predictive value; PPV: positive-predictive value; SEN: sensitivity; SPE: specificity; TM: topic mapping.

The BRPRD sample data evaluated the MV-LDA model. The performance of the MV-LDA model is shown in Figure 3. Each model showed higher specificity and NPV for some topics, with the NPV reaching more than 90%, and the sensitivity being the highest at a TM threshold of 1. As the threshold value declined, the sensitivity decreased, the specificity and PPV increased, and the NPV showed no relevant change. When the number of topics increased, the sensitivity increased greatly, but the specificity, PPV, and NPV changed little.

Taking all factors (sensitivity, specificity, PPV, NPV, and Youden’s index) into consideration, we set a cutoff of ≥1 TM anomaly scoring as the threshold for our MV-LDA detection model. The results showed a high sensitivity of 81.8% and a moderate specificity of 47.4%, and the PPV and NPV were 14.5% and 96.0%, respectively. These findings indicate that under the best performance parameter setting, we can find 1208 of 1476 inappropriate prescriptions.

Our model evaluation results revealed that the MV-LDA model had a better ability to detect inappropriate prescriptions when the TM threshold was set to 1. However, for a better understanding of the robustness of the results at this parameter setting, we performed a sensitivity analysis, repeating the experiments with separate sampling proportions of 90%, 70%, 50%, 30%, and 10%. Table 3 presents the findings. There were no relevant differences between the two experiments.

**Table 3:** Sensitivity analysis for multiview latent Dirichlet allocation with topic mapping detection methods (threshold=1).

Sampling proportion	TP^a^, n (%)	FP^b^, n (%)	FN^c^, n (%)	TN^d^, n (%)	SEN^e^ (%)	SPE^f^ (%)	PPV^g^ (%)	NPV^h^ (%)	Youden’s index	AUROC^i^
90%	1073 (7.9)	5752 (42.6)	249 (1.8)	6435 (47.6)	81.2	47.2	14.3	95.9	14.3	0.689
70%	823 (7.9)	4446 (42.7)	179 (1.7)	4954 (47.6)	82.1	47.3	14.2	96.1	14.2	0.695
50%	570 (7.6)	3235 (43.1)	143 (1.9)	3562 (47.4)	79.9	47.6	13.8	95.8	13.8	0.686
30%	383 (8.6)	1958 (43.9)	93 (2.1)	2030 (45.5)	80.5	49.1	15.9	95.5	15.9	0.705
10%	109 (7.3)	622 (41.9)	21 (1.4)	734 (49.4)	83.8	45.9	12.9	96.7	12.9	0.693

^a^TP: true positive.

^b^FP: false positive.

^c^FN: false negative.

^d^TN: true negative.

^e^SEN: sensitivity.

^f^SPE: specificity.

^g^PPV: positive-predictive value.

^h^NPV: negative-predictive value.

^i^AUROC: area under the receiver operating characteristic curve.

## Discussion

### Principal Findings

The study drew upon the data of almost 45 million prescriptions obtained from the CMNRUD database (between October and December 2016). It then used the MV-LDA combination of TM anomaly detection and LDA topic modeling to build a model for detecting the inappropriate use of prescription medication. The model had a sensitivity of 81.8% and a specificity of 47.4% with 45 topics, and it had an anomaly threshold of 1 and showed stability in the sensitivity analysis. The topics that were already built into our study included most disorders, and the topics that were generated included noncommunicable diseases, such as cardiovascular diseases, which appeared in the largest proportions, consistent with clinical practice. The model also accommodated the tendency for many disorders to be seen in winter. For instance, upper respiratory infections, fever, and acute bronchitis were determined to be highly probable in winter. It took only 3.5 hours to generate the topics and only seconds to detect an anomaly, much quicker than the system of manual review or knowledge-based prescription review.

### Limitations

The present work has several limitations. The MV-LDA model has more features than were used in this study, including usage, dosage, cost, and even laboratory test results, when available. Moreover, it was challenging to clean accessional variables from the data source. For example, there are multiple modes of recording dose packaging because the composition and dosage forms of medicines differ from each other. Because of the difficulties noted above, the first limitation is that our method ignored the medication’s usage and dosage and only addressed the medication itself when building the MV-LDA model and validating the results. Second, the current model is still not supported for indicating the specific medication but tells us which prescriptions do not comply with the prescription review criteria. Besides, limited by the failure to obtain a labeled training dataset, the current model is not able to classify prescription misuse by criteria, such as the absence of proper indications, violation of clinical guidelines, and misuse of dosage, and can only detect the appropriateness of prescriptions. This study is also limited by the diverse structure of the model training and evaluation database and a minor overlap between the datasets used. However, we thought that a minor overlap of the data might not be associated with a major change in the results. Meanwhile, the two databases showed a commonality in their treatment patterns, and this, in fact, could be a topic for exploratory research on methodology. While this study focused on the development of a model, in the future, we will address a diverse range of parameters to determine the most effective MV-LDA model for detecting prescription misuse.

### Comparison With Prior Work

Studies, such as those encouraging regular medication review and introducing automated information systems [47,48], have been conducted with the aim of controlling the inappropriate use of medications in China. However, the increasing number of new drugs entering the market, delays in updating the databases, and insufficient knowledge of medications all raise the probability of nonideal use [49]. Knowledge-based and experience-based software has relevant limitations, including efficiency constraints. However, data mining techniques are customizable and can identify inappropriate prescriptions. For example, association rule mining has been used to find inappropriate prescriptions by calculating the co-occurrence of medications and diseases, resulting in a sensitivity of 75.9% and a specificity of 89.5% [42,43]. These methods however do have disadvantages, including inefficiency in the generation of candidate item sets because they require vast data sources and the frequent scanning of databases.

Furthermore, these methods often fail to explore latent structures and are prone to making spurious associations that can mislead clinical practitioners. Besides, in a previous study, a model combining natural language processing with guidelines based on expert knowledge was used to detect medication overuse, and it showed degrees of sensitivity and specificity that were similar to those in our study [50]. Despite using different data sources and operating under diverse study conditions, we noted a higher sensitivity as compared with the association rule mining method. Although we failed to obtain a higher PPV, which is strongly related to the prevalence of inappropriate prescriptions, we think the MV-LDA model is suitable for preliminary screening and can be an alternative detection method, allowing clinical practice to flag potentially inappropriate prescriptions for manual review. Such a step could save a large amount of working time and reduce labor intensity.

A topic model is a multiple machine learning method and is used to reveal the semantics in the body of the text. With its advantages of topic extraction and model expansibility, LDA has become a commonly used topic modeling method. It was first used to extract underlying semantics and was then optimized to become a robust means of text mining analysis for social media [51-53]. Recently, topic modeling methods, particularly LDA, have been used for both structured and unstructured clinical data [21,26,54-56]. Several studies have attempted to scale the efficiency of LDA’s topic generation. The symptom-herb-diagnosis topic model, which was proposed to determine the association between treatment with Chinese medicine and diabetes, can be used to find herbs to treat specific symptoms [55]. Multiple-channel LDA [57] focused on the support system for clinical decisions and based itself on a similar concept that the coupling of diagnoses and medications reflects the health status of patients at the time of seeing a doctor. However, we were not able to obtain a piece of well-recorded contextual information or additional information, but we could leverage two variables (diagnosis and medication) and realize the aim of the study. Besides, given the shortcomings of miscellaneous algorithms and more extended calculations of an LDA-based model, various measures were taken to improve the MV-LDA algorithm in our project, which is not the focus of this study. The multiview topic modeling approach used here has been previously tested in different languages [34]. This allowed us to take both medications and diagnoses into consideration simultaneously. We leveraged these advantages and processed only half of the data used in a previous study to determine the association between diagnoses and medications for each prescription [58]. Prescriptions in our study were considered the equivalent of articles in that previous study, reflecting the particular situation of those patients. The topics and their allocations were consistent with clinical practice, providing proof of the robustness of our method.

### Conclusions

Our MV-LDA model can train the distribution of diagnosis-medication topics from a large number of prescriptions and can detect the potentially inappropriate use of prescription medications when combined with the TM method. Considering its mediocre specificity and moderate sensitivity, this model can be used as a primary screening tool and will likely complement and improve manual review. The model still needs more extension of views (introduction of more variables) to make full use of the information in the prescription and further improve the ability to identify prescription misuse.

## Appendix

Multimedia Appendix 1 Confusion matrix of model evaluation (multiview latent Dirichlet allocation model versus experts).

Multimedia Appendix 2 Example results of multiview latent Dirichlet allocation topic modeling (K=30).

## Abbreviations

BRPRD: Beijing Regional Prescription Review Database
CMNRUD: Chinese Monitoring Network for the Rational Use of Drugs
LDA: latent Dirichlet allocation
MV-LDA: multiview latent Dirichlet allocation
NPV: negative-predictive value
PPV: positive-predictive value
TM: topic mapping
