# Supplementary material for: A Multiview Model for Detecting the Inappropriate Use of Prescription Medication: Machine Learning Approach
Source: JMIR Med Inform. 2020 Jul 6;8(7):e16312. doi: 10.2196/16312 (PMC7381037; doi:10.2196/16312)
Supplement: Multimedia Appendix 1 [file medinform_v8i7e16312_app1.docx]

Appendix 1 Confusion matrix of model evaluation (MV-LDA model versus experts)^a^

|  | | **Experts** | |
| --- | --- | --- | --- |
|  |  | Inappropriate | Appropriate |
| **MV-LDA model** | Potential Inappropriate | TP | FP |
|  | Appropriate | FN | TN |

^a^ TP: true positive; FP: false positive; FN: false negative; TN: true negative.
