# Supplementary material for: A Multiview Model for Detecting the Inappropriate Use of Prescription Medication: Machine Learning Approach
Source: JMIR Med Inform. 2020 Jul 6;8(7):e16312. doi: 10.2196/16312 (PMC7381037; doi:10.2196/16312)
Supplement: Multimedia Appendix 2 [file medinform_v8i7e16312_app2.docx]

Appendix 2 Example results of MV-LDA topic modeling (K=30)

| # 1 | Medication | Cefixime:0.0553, Child Chiqiaoqingre granules:0.0528, Pudilan Xiaoyan oral liquid:0.0465, Kai hou jian Pen wu ji:0.04, Jubei Heji:0.0345, Xiyanping injection:0.0251, Pidomomod:0.0239, Ribavirin:0.0211, Bairui Granules:0.02 |
| --- | --- | --- |
|  | Diagnosis | Acute tonsillitis:0.2258, Acute asthmatic bronchitis:0.0768, Herpes zoster:0.0728, Herpetic angina:0.0399, Hand, foot and mouth disease:0.0305, Tonsillitis:0.0299, Viral rash:0.0159, Acute otitis media:0.0148, Stomatitis:0.0122 |
| # 2 | Medication | aspirin:0.1796, Clopidogrel:0.1232, Rosuvastatin:0.1112, Metoprolol:0.0664, Isosorbide mononitrate:0.057, Trimetazidine:0.0279, Bisoprol:0.0192, Simvastatin:0.0167, Pivastatin:0.0159 |
|  | Diagnosis | Hyperlipidemia:0.3593, Atherosclerosis:0.0632, Coronary stent implantation status:0.0282, Hypertensive heart disease:0.0173, Angina pectoris:0.0167, Hemiplegia:0.0063, Hypertension I:0.0061, Heart infarction:0.0056, Unstable angina:0.0045 |
| # 3 | Medication | Glucosamine:0.0418, Loxoprofen:0.0391, Celecoxib:0.0318, Eperisone:0.0303, Lofen:0.03, Meloxicam:0.0271, Diclofenac:0.0221, Horse tetanus immunoglobulin:0.0217, Aceclofenac:0.0215 |
|  | Diagnosis | Head trauma:0.0659, Spine disease:0.0497, Injure:0.0323, Soft tissue disorder:0.0291, Knee pain:0.0201, Hand injury:0.017, Periarthritis of shoulder:0.016, Fasciitis:0.0156, Skin laceration:0.0155 |
| # 4 | Medication | Calcitriol:0.0646, Mecobalamin:0.0351, Maizhiling tablets:0.0348, Glucosamine:0.032, Cold and clear granules:0.0249, Alfacalcidol:0.0214, Alendronic acid:0.0207, Liu Wei Di Huang Wan:0.0172, Ginkgo leaf:0.0158 |
|  | Diagnosis | Cerebrovascular disease:0.1545, Cold:0.1006, Joint pain:0.0767, Osteoarthritis:0.058, Peripheral neuropathy:0.0351, Unspecified hyperlipidemia - other diseases:0.0338, Arteriosclerosis:0.0251, Arthropathy - other diseases:0.0247, Neuritis:0.0161 |
| # 5 | Medication | Prednisone:0.1009, Hydroxychloroquine:0.0605, Methylprednisolone:0.0472, Leflunomide:0.0383, Total Glucosides of White Paeony :0.0333, Methotrexate:0.0292, Alfacalcidol:0.0267, Cefprozil:0.0252, Prednisolone:0.0243 |
|  | Diagnosis | Rheumatoid arthritis:0.1028, Palpitation:0.0926, Hypertension III:0.0778, Skin infection:0.05, Unspecified allergic rhinitis - other diseases:0.0454, Weak:0.0332, Thyroid nodules:0.0293, Viral upper respiratory tract infection:0.0202, Hemorrhoid:0.02 |
| # 6 | Medication | Yeast:0.0864, Recombinant human interferon alpha 2b:0.0596, Diosmin:0.0365, Compound polyethylene glycol electrolyte:0.0285, Thymosin α1:0.0265, Ubumex:0.0203, Puji hemorrhoids suppository:0.0177, Multidimensional element:0.0171, Thymosin:0.0157 |
|  | Diagnosis | Normal pregnancy supervision:0.0787, Mixed hemorrhoid:0.0604, Hypothyroidism:0.0519, Colitis:0.0333, Thyroid malignancy:0.025, Massive Cerebral Infarction:0.0235, Posterior circulation ischemia:0.0195, Human papillomavirus infection:0.0165, Anal fissure:0.0157 |
| # 7 | Medication | Sterilized water for injection:0.1094, Vitamin B1:0.0613, rabies vaccine:0.0579, Adenosine cobalamin:0.0413, Diclofenac:0.0317, Gabapentin:0.0282, Ginkgo biloba extract:0.0242, Ginkgo leaf:0.0215, Tramado:0.0194 |
|  | Diagnosis | Vertigo:0.1019, Tinnitus:0.0651, Ureteral calculi:0.0377, Otitis externa:0.0372, Urolithiasis:0.0325, Renal colic:0.0318, Kidney stones:0.031, Cerebral arteritis:0.0285, Ankylosing spondylitis:0.0273 |
| # 8 | Medication | Acarbose:0.1367, Insulin glargine:0.0618, 70% (low-protein insulin aspart) 30% (aspart insulin) mixed insulin:0.0613, Glimepiride:0.0556, Riglinay:0.0519, 70% (low protamine human insulin) 30% (human insulin) mixed insulin:0.0509, Gliczit:0.0413, Pioglitazone:0.0326, Voglibose:0.0259 |
|  | Diagnosis | Diabetic nephropathy:0.0259, Hypoglycemia:0.021, Type 2 diabetes with complications:0.0166, Diabetic peripheral neuropathy:0.0141, Type 2 diabetes with multiple complications:0.014, Diabetic retinopathy:0.0132, Type 2 diabetic nephropathy:0.0113, Diabetic peripheral vascular disease:0.0077, Diabetic neuropathy:0.0059 |
| # 9 | Medication | Ambroxol:0.0399, Dexamethasone:0.023, Pantoprazole:0.0219, Lansoprazole:0.0207, Omeprazole:0.0202, Xingnaojing injection:0.0193, Moxifloxacin:0.018, Mannitol:0.0163, Furosemide:0.016 |
|  | Diagnosis | Lung infection:0.188, Dizziness and vertigo:0.0889, Cerebral infarction sequela:0.0545, Cardiac insufficiency:0.0542, Lacunar infarction:0.0358, Hypokalemia:0.0245, Hemorrhage:0.0221, Malignant tumor:0.0146, Cerebrovascular accident:0.0138 |
| # 10 | Medication | Betastatin:0.0612, Flunarizine:0.0349, Olaxitan:0.0334, Nimodipine:0.0291, Niger hornwood:0.028, Sodium valproate:0.0269, Pulse blood capsule:0.0229, Yang serum brain particles:0.0184, Dengsheng Shengmai Capsule:0.0181 |
|  | Diagnosis | Epilepsy:0.0909, Cerebral artery insufficiency:0.0707, Vertiginous syndromes:0.0386, Heart failure:0.0359, Stroke:0.0325, Neuralgia:0.0309, Cerebrovascular insufficiency:0.0262, Vertebral-basal artery insufficiency:0.0255, Prescribe again at the health facility:0.0196 |
| # 11 | Medication | Dydrogesterone:0.0383, Nifuratel/mycin:0.0344, Estradiol valerate:0.0299, Urinary gonadotropin:0.0275, Bao Fu Kang Suppository:0.0207, Chorionic gonadotropin:0.0199, Vitamin E:0.0193, Lactobacillus:0.0173, Red nuclear women's cleansing lotion:0.0168 |
|  | Diagnosis | Female infertility:0.1091, Vaginitis:0.0915, Irregular menstruation:0.0705, Confirm pregnancy:0.051, Inflammatory disease of cervix uteri:0.0406, Female pelvic inflammatory disease:0.0246, Threatened abortion:0.0218, Chronic vaginitis:0.0192, Artificial abortion:0.0184 |
| # 12 | Medication | Mosapride:0.0585, Trimebutine:0.045, Omeprazole:0.0437, Hydrotalcite:0.0336, Esomeprazole:0.0321, Lansoprazole:0.0277, Multi-enzyme:0.0263, Itobili:0.025, Rebate:0.0229 |
|  | Diagnosis | Gastrointestinal dysfunction:0.068, Breast malignancy:0.0623, Erosive gastritis:0.0515, Pulmonary malignant tumor:0.0476, Gastroesophageal reflux disease:0.0383, Intestinal dysfunction:0.0369, Esophagitis:0.0215, Gastric malignancy:0.0184, Duodenal ulcer:0.0162 |
| # 13 | Medication | Vitamin C:0.0608, Electrolyte replenisher:0.0472, Montmorillonite:0.0385, Xiyanping injection:0.0316, Cefazolin:0.0313, Oral rehydration salt:0.0303, Vitamin B6:0.028, Ribavirin:0.0274, Glucose/sodium chloride:0.0273 |
|  | Diagnosis | Acute gastroenteritis:0.0844, Infectious fever:0.0844, Diarrhea:0.0777, Chest pain:0.0584, Chronic obstructive pulmonary disease:0.0576, Chest tightness:0.0457, Enteritis:0.0378, Acute laryngitis:0.0304, Bacterial infection:0.0266 |
| # 14 | Medication | Cefaclor:0.0663, Adefovir dipivoxil:0.0484, Cefixime:0.0455, Ursodeoxycholic acid:0.0392, Silibinin:0.0355, Diammonium glycyrrhizinate:0.0335, Bicyclic alcohol:0.0272, Polyene phosphatidylcholine:0.0272, Lamivudine:0.0265 |
|  | Diagnosis | Abnormal liver function:0.0659, Chronic hepatitis B:0.0651, Cirrhosis:0.0509, Fatty liver disease:0.0449, Liver disease:0.0373, Chronic hepatitis:0.0337, Viral hepatitis type b:0.0277, Hepatic insufficiency:0.0222, Hepatic malignant tumor:0.0222 |
| # 15 | Medication | Sodium silicate:0.1146, Ofloxacin:0.0616, Praprofen:0.0606, Recombinant bovine basic fibroblast growth factor:0.0523, Tobramycin/dexamethasone:0.0406, Tobramycin:0.0325, Fluoromethane:0.0257, Compound tropicamide:0.0244, Recombinant human epidermal growth factor:0.0237 |
|  | Diagnosis | Dry eye syndrome:0.0749, Refractive error:0.0589, Cataract:0.0503, Keratitis:0.0314, Glaucoma:0.03, Chronic superficial gastritis:0.0232, Chronic conjunctivitis:0.018, Vitreous opacity:0.0176, Corneal foreign body:0.0135 |
| # 16 | Medication | Compound methoxyphenamine:0.0852, Budesonide:0.0684, Suhuang Zhike Capsule:0.0373, Budesonide Formoterol:0.0329, Azithromycin:0.0298, Salmeterol:0.0294, Tiotropium bromide:0.027, Terbutaline:0.0262, Salbutamol:0.0261 |
|  | Diagnosis | Asthmatic bronchitis:0.1212, Asthma:0.117, Bronchial Asthma:0.0578, Allergic purpura:0.0381, Bronchial asthma, non-critical:0.0371, Acute exacerbation of chronic bronchitis:0.0291, Respiratory disease:0.0273, Cough variant asthma:0.025, Allergic rhinitis with asthma:0.0183 |
| # 17 | Medication | Pantoprazole:0.0669, Omeprazole:0.0629, Anisodamine:0.0445, Compound Lactobacillus acidophilus:0.0352, Vitamin C:0.0297, Potassium chloride:0.0296, Glucose/sodium chloride:0.0289, Bifidobacterium/Lactobacillus acidophilus/Enterococcus cocci:0.0268, glycerin:0.0247 |
|  | Diagnosis | Gastroenteritis:0.0973, Reflux esophagitis:0.0964, Vomiting:0.0598, Stomach ulcer:0.0499, Intestinal flora alternation:0.0361, Acute gastritis:0.0313, Peptic ulcer:0.03, Gastrointestinal bleeding:0.0288, Bronchiectasis with infection:0.0169 |
| # 18 | Medication | Pudilan Xiaoyan oral liquid:0.0487, Cefuroxime:0.0443, Cefaclor:0.04, Cefixime:0.0361, Lanqi oral solution:0.0309, Phenol memin:0.0295, Amoxicillin/clavulanic acid:0.0272, Child Chiqiaoqingre granules:0.0257, Pediatric Chai Gui Antipyretic Granules:0.0235 |
|  | Diagnosis | Acute suppurative tonsillitis:0.0711, Upper respiratory disease:0.0382, Rash:0.0217, Pneumoperitoneum:0.0156, Thrombocytopenia:0.015, Acute bronchiolitis:0.0059, Unspecified acute bronchiolitis - other diseases:0.0052, Influenza:0.0052, Tumor:0.0047 |
| # 19 | Medication | Alprazolam:0.0731, Flutethiophene / melitracen:0.066, Clonazepam:0.0447, Olanzapine:0.0406, Paroxetine:0.0373, Zolpidem:0.0348, Essie Puplan:0.0341, Sertraline:0.0323, Diazepam:0.0294 |
|  | Diagnosis | Anxiety state:0.1516, Primary or maintenance sleep disorder insomnia:0.1073, Primary or maintenance sleep disorders insomnia - other diseases:0.0671, Depression:0.0565, Depression:0.0451, Anxiety disorder:0.0345, Schizophrenia:0.0308, Mental disorder:0.0208, Neurosis:0.0129 |
| # 20 | Medication | Ketotifen:0.0857, Chlorpheniramine:0.0477, Monteluk:0.0433, Pulmonary cough mixture:0.0347, Cefixime:0.03, Cefprozil:0.0269, Ambroxol:0.0244, Pidomomod:0.0237, Acetylcysteine:0.0234 |
|  | Diagnosis | Mycoplasma infection:0.0573, Acute bronchitis:0.0453, Rheumatoid arthritis:0.0279, Unspecified acute tonsillitis - other diseases:0.026, Allergic cough:0.0194, Tracheobronchitis:0.0187, Autoimmune disease:0.0187, Angina:0.0147, Acute angina:0.014 |
| # 21 | Medication | Eucalyptol Limonene and Pinene:0.0815, Desloratadine:0.0791, Loratadine:0.0473, Methimazole:0.0433, Cetirizine:0.0384, Azelastine:0.0369, Biyuan Tongyu Granules:0.0326, Trinity:0.0233, Tongqiao rhinitis granules:0.0201 |
|  | Diagnosis | Rhinitis:0.0832, Otitis media:0.0625, Sinusitis:0.0575, Chronic rhinitis:0.0564, Hyperthyroidism:0.0463, Chronic sinusitis:0.0436, Breast hyperplasia:0.0406, Psoriasis:0.0331, Acute nasopharyngitis:0.0273 |
| # 22 | Medication | Desloratadine:0.0631, Levocetirizine:0.0558, Ebastine:0.0442, Dyne:0.0417, Citalopic acid:0.0351, Halabisone:0.0329, Triamcinolone acetonide/econazole:0.0266, Hydrocortisone:0.0252, Cetirizine:0.0227 |
|  | Diagnosis | Dermatitis:0.1441, Hemorrhoids:0.076, Allergic dermatitis:0.0738, Urticaria:0.0712, Seborrheic dermatitis:0.0313, Nausea and vomiting:0.0267, Folliculitis:0.0258, Vitiligo:0.0218, Acute urticaria:0.0186 |
| # 23 | Medication | Tanloxin:0.0983, Cefdinir:0.0689, Finasteride:0.0584, Cefixime:0.0293, Hot granule:0.0292, Doxazosin:0.0168, Yinhua Biyanling Tablets:0.0147, Ningbitai capsule:0.0147, Longbishu Jiaonang:0.014 |
|  | Diagnosis | Prostatic hyperplasia:0.1575, Urinary tract infection:0.1496, Coronary heart disease:0.073, Heart disease:0.0675, Urethral infection:0.0435, Prostatitis:0.0347, Hematuria:0.0201, Irritable bowel syndrome:0.0157, Cerebrovascular disease sequelae:0.0142 |
| # 24 | Medication | Budesonide:0.1074, Azithromycin:0.0993, Terbutaline:0.0621, Glucose/sodium chloride:0.0397, Ipratropium bromide:0.0391, Xiyanping injection:0.0239, Ambroxol:0.0199, Yan Huning:0.0191, Phenolmethine:0.0177 |
|  | Diagnosis | Pneumonia:0.2184, Bronchopneumonia:0.1213, tracheitis:0.0344, Asthmatic bronchopneumonia:0.0181, Tonsil pharyngitis:0.015, Unexplained convulsion:0.0083, Perivascular inflammation:0.0065, Acute tracheobronchitis:0.0056, Febrile seizure:0.0033 |
| # 25 | Medication | Lanqi oral solution:0.0648, Cefuroxime:0.0565, Atticacaine/adrenalin:0.0508, Cefaclor:0.0448, Cefprozil:0.0434, Roxithromycin:0.0375, Dexamethasone:0.0305, Ornidazole:0.0276, Clindamycin:0.026 |
|  | Diagnosis | Chronic pharyngitis:0.1326, Acute pharyngitis:0.0709, Systemic lupus erythematosus:0.066, Laryngitis:0.0445, Tooth impaction:0.0386, Periodontitis:0.0205, Pulpitis:0.02, Lymphadenitis:0.0192, Chronic laryngitis:0.0184 |
| # 26 | Medication | Sodium bicarbonate:0.0565, Recombinant human erythropoietin (CHO cells):0.0515, L-carnitine:0.0399, Jinshuibao capsule:0.0396, Calcitriol:0.0353, Low molecular weight heparin:0.0346, Benzobromarone:0.0288, Polysaccharide iron complex:0.0271, Compound α-keto acid:0.0271 |
|  | Diagnosis | Anemia:0.059, Hyperuricemia:0.0533, Chronic renal insufficiency:0.0513, Chronic renal failure:0.0475, Renal anemia:0.0432, Gout:0.0403, Renal osteopathy:0.0393, Nephrotic syndrome:0.0368, Renal insufficiency:0.0345 |
| # 27 | Medication | Eryphilin:0.0383, Vitamin D:0.036, Vitamin AD:0.0359, Benzalkonium chloride:0.0334, penicillin:0.0286, Clindamycin:0.0284, Compound polymyxin B:0.0284, Rehabilitation new fluid:0.0267, Dobs:0.0263 |
|  | Diagnosis | Pain:0.0758, Chronic prostatitis:0.0644, Parkinson's Disease:0.0536, Gonarthrosis:0.0262, Rickets:0.0235, Gouty arthritis:0.0224, Hair loss:0.0222, Vitamin D deficiency:0.0222, Glans dermatitis:0.0172 |
| # 28 | Medication | Nifedipine:0.0894, Levamlodipine:0.0773, Metoprolol:0.061, Irbesartan:0.0578, Valsartan:0.0539, aspirin:0.0492, Bisoprol:0.0446, Irbesartan/hydrochlorothiazide:0.0384, Felodipine:0.0349 |
|  | Diagnosis | Essential hypertension:0.0309, Coronary heart disease arrhythmia:0.0167, Coronary artery stenosis:0.0125, Malignant hypertension:0.0053, Other parasympathetic inhibitor anticholinergic drugs - other diseases:0.0006, Harmful effects of salicylates - other diseases:0.0004, Bone hypertrophy - other diseases:0.0001, Maxillary malignant tumor:0, Superior mesenteric artery aneurysm:0 |
| # 29 | Medication | Clarithromycin:0.1146, Pantoprazole:0.0588, Amoxicillin:0.054, Rehabilitation new fluid:0.0508, Omeprazole:0.0497, Mosapride:0.0482, Lansoprazole:0.0455, Dakronin:0.0407, Amoxicillin/clavulanic acid:0.0346 |
|  | Diagnosis | Helicobacter pylori infection:0.155, Lumbar disc herniation:0.1093, Neurasthenia:0.0219, Sjogren syndrome:0.0212, Chronic colitis:0.0183, Connective tissue disease:0.0166, Duodenal ulcer:0.0116, Mixed cervical spondylosis:0.0105, Heart valve disease:0.0091 |
| # 30 | Medication | Aspirin:0.0652, Clopidogrel:0.0629, Trimetazidine:0.0471, Atorvastatin:0.0462, Isosorbide mononitrate:0.0403, Spironolactone:0.0398, Rosuvastatin:0.0362, Steady heart particle:0.0271, Warfarin:0.0259 |
|  | Diagnosis | Constipation:0.1173, Arrhythmia:0.091, Chronic bronchitis:0.0862, Hypertension II:0.0607, Atrial fibrillation:0.0408, Chronic coronary insufficiency:0.0328, Cholecystitis:0.0295, Coronary atherosclerosis:0.0179, Gallstone:0.0161 |
